# The genome sequence of common box, *Buxus sempervirens *L. (Buxaceae)

**DOI:** 10.12688/wellcomeopenres.23267.1

**Published:** 2024-11-20

**Authors:** Maarten J. M. Christenhusz, Michael F. Fay, Royal Botanic Gardens Kew Genome Acquisition Lab, Darwin Tree of Life Barcoding collective, Plant Genome Sizing collective, Wellcome Sanger Institute Tree of Life Management, Samples and Laboratory team, Wellcome Sanger Institute Scientific Operations: Sequencing Operations, Wellcome Sanger Institute Tree of Life Core Informatics team, Tree of Life Core Informatics collective, Darwin Tree of Life Consortium

**Affiliations:** 1 Royal Botanic Gardens Kew, Richmond, England, UK; 2 Curtin University, Perth, Western Australia, Australia

**Keywords:** Buxus sempervirens, common box, genome sequence, chromosomal, Buxales

## Abstract

We present a genome assembly from an individual *Buxus sempervirens* (common box; Streptophyta; Magnoliopsida; Buxales; Buxaceae). The genome sequence has a total length of 676.70 megabases. Most of the assembly (99.56%) is scaffolded into 14 chromosomal pseudomolecules. The plastid genome assembly is 150.93 kilobases in length, and 8 mitochondrial sequences were also assembled.

## Species taxonomy

Eukaryota; Viridiplantae; Streptophyta; Streptophytina; Embryophyta; Tracheophyta; Euphyllophyta; Spermatophyta; Magnoliopsida; Mesangiospermae; Buxales; Buxaceae; *Buxus*; *Buxus sempervirens* L. (Buxaceae) (NCBI:txid4002).

## Background

*Buxus sempervirens* L., known as box or common box, is an evergreen shrub or small tree up to 10 m tall, with opposite, yellowish green oval leaves. It produces fragrant, yellowish flowers in early spring, followed by three-lobed capsules holding three or six black seeds ( Bean, 1976). The foliage has a particular smell which some people find unpleasant. The species occurs from southern England to North Africa and east to the Caucasus and Iran ( POWO, 2024), but it is commonly grown in gardens and planted individuals can persist or spread into nearby forests both within its natural range or outside it in northern and eastern Europe, North America, New Zealand and locally in the Andes and eastern Himalayas ( POWO, 2024). Natural stands are confined to chalk or limestone soils, often in the understory of taller beech forests. In Britain, these are at least mostly in south-east England. The stands at Box Hill are perhaps best known ( Bean, 1976); the sequenced sample came from this site. Other English place names thought to be based on the name of the species (e.g. Bix Bottom, Oxfordshire) may indicate that it was previously more widespread. On Box Hill, accompanying species include *Taxus baccata* (common yew), another indicator of chalk soils.

In Britain, box has been cultivated since Roman times, as attested to by sprigs of *B. sempervirens* found in British-Roman settlements ( Lodwick, 2017). The species is popular in formal gardens used for topiary and hedging, due to its evergreen nature, small leaves and tolerance to frequent pruning. However, use of *Buxus* L. in horticulture is declining in western Europe due to the increase of invasive pests, such as the box tree caterpillar ( *Cydalima perspectalis*), box sucker ( *Psylla buxi*), mussel scale ( *Lepidosaphes ulmi*), red spider mite ( *Tetranychus urticae*), and fungal diseases, including box blight ( *Calonectria* spp.), volutella blight ( *Pseudonectria buxi*), box rust ( *Puccinia buxi*), leaf spot ( *Macrophoma candollei*) and *Phytophthora* root rot ( AFPP, 2018; Kenis *et al.*, 2013; Mitchell *et al.*, 2018). The box tree moth ( *Cydalima perspectalis*) is a particularly problematic pest that was recently introduced from East Asia. Together with invasive box blight (introduced in the 1990s), *C*. *perspectalis* is a major threat to native stands of *B. sempervirens* ( Kenis *et al.*, 2013; Mitchell *et al.*, 2018; Salisbury *et al.*, 2012).

The slow growth of *B. sempervirens* renders its wood very hard, heavy and grain-free, which makes it useful for cabinetry, musical instruments (flutes, oboes), engraving, tool handles, wood inlays and, of course, decorative boxes ( Grigson, 1974). Due to the decline in population sizes and the absence of large specimens, however, these uses are mostly historical. The plant has also been used in various rituals, from burial tokens in Roman graves in Britain ( Lodwick, 2017), to being used as a substitute for palm leaves or olive branches on Palm Sunday during Catholic traditions in Germanic countries ( De Cleene & Lejeune, 2000).

Medical properties have not been widely tested, but compounds identified include steroidal alkaloids (e.g. cyclobuxine), tannins, oils and flavonoids (e.g. galactobuxin), and the species may have many potentially beneficial compounds ( Ahmed *et al.*, 1988; Atta-ur-Rahman *et al.*, 1991; Palchykov *et al.*, 2020). It was formerly used as a substitute for quinine to treat malaria, but because of its many negative side-effects this usage fell out of favour ( Neves *et al.*, 2009).

The sample sequenced here was taken from a plant cultivated at the Royal Botanic Gardens, Kew, of known wild source (Box Hill, Surrey). The high-quality genome sequence will be useful for studies on population genetics and disease resistance in this species. It may also help understand the toxicology of this species and potentially aid in the discovery of medicinally useful compounds.

## Genome sequence report

**Figure 1.**
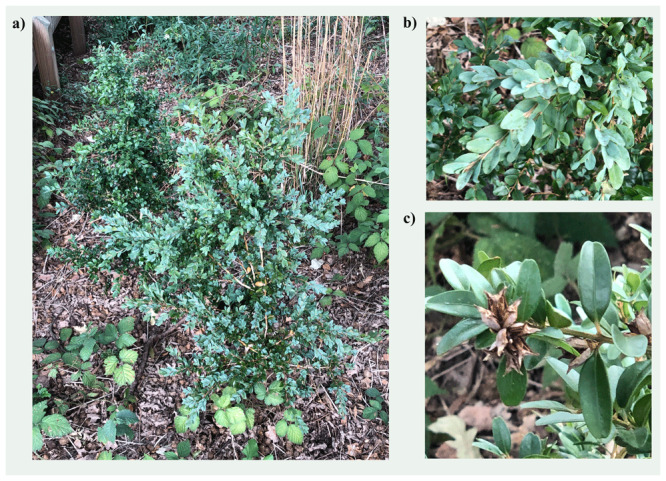
Photographs of the *Buxus sempervirens* (drBuxSemp1) specimen from which samples were taken for genome sequencing.

The genome of a specimen of *Buxus sempervirens* ( Figure 1) was sequenced using Pacific Biosciences single-molecule HiFi long reads, generating a total of 22.84 Gb (gigabases) from 1.64 million reads. The coverage was estimated to be approximately 31-fold. Using flow cytometry, the genome size (1C-value) was estimated to be 0.91 pg, equivalent to 890 Mb.

Primary assembly contigs were scaffolded with chromosome conformation Hi-C data, which produced 111.85 Gb from 740.74 million reads, yielding an approximate coverage of 165-fold. Specimen and sequencing details are summarised in Table 1.

**Table 1.** Specimen and sequencing data for *Buxus sempervirens*.

Project information			
**Study title**	Buxus sempervirens		
**Umbrella BioProject**	PRJEB53560		
**Species**	*Buxus sempervirens*		
**BioSample**	SAMEA7522626		
**NCBI taxonomy ID**	4002		
Specimen information			
**Technology**	**ToLID**	**BioSample accession**	**Organism part**
**PacBio long read sequencing**	drBuxSemp1	SAMEA7522649	leaf
**Hi-C sequencing**	drBuxSemp1	SAMEA7522649	leaf
**RNA sequencing**	drBuxSemp1	SAMEA7522648	leaf
Sequencing information			
**Platform**	**Run accession**	**Read count**	**Base count (Gb)**
**Hi-C Illumina NovaSeq 6000**	ERR9866448	7.41e+08	111.85
**PacBio Sequel IIe**	ERR9863247	1.64e+06	22.84
**RNA Illumina NovaSeq 6000**	ERR10378016	5.94e+07	8.97
**RNA Illumina NovaSeq 6000**	ERR10378015	6.12e+07	9.24

Assembly errors were corrected by manual curation, including 49 missing joins or mis-joins and five haplotypic duplications. This reduced the assembly length by 0.99% and the scaffold number by 66.44%, and increased the scaffold N50 by 0.97%. The final assembly has a total length of 669.50 Mb in 91 sequence scaffolds, with 408 gaps, and a scaffold N50 of 48.9 Mb ( Table 2).

**Table 2.** Genome assembly data for *Buxus sempervirens*, drBuxSemp1.3.

Genome assembly		
Assembly name	drBuxSemp1.3	
Assembly accession	GCA_947561395.3	
*Accession of alternate haplotype*	*GCA_947561485.2*	
Span (Mb)	676.70	
Number of contigs	706	
Contig N50 length (Mb)	3.2	
Number of scaffolds	258	
Scaffold N50 length (Mb)	48.9	
Longest scaffold (Mb)	73.12	
Assembly metrics *		*Benchmark*
Consensus quality (QV)	63.5	*≥ 50*
*k*-mer completeness	100.0%	*≥ 95%*
BUSCO **	C:99.0%[S:95.5%,D:3.5%], F:0.4%,M:0.6%,n:1,614	*C ≥ 95%*
Percentage of assembly mapped to chromosomes	99.56%	*≥ 95%*
Organelles	Plastid assembly and 8 mitochondrial sequences.	*complete single alleles*

* Assembly metric benchmarks are adapted from column VGP-2020 of “Table 1: Proposed standards and metrics for defining genome assembly quality” from Rhie *et al.* (2021). ** BUSCO scores based on the embryophyta_odb10 BUSCO set using version 5.4.3. C = complete [S = single copy, D = duplicated], F = fragmented, M = missing, n = number of orthologues in comparison.

**Figure 2.**
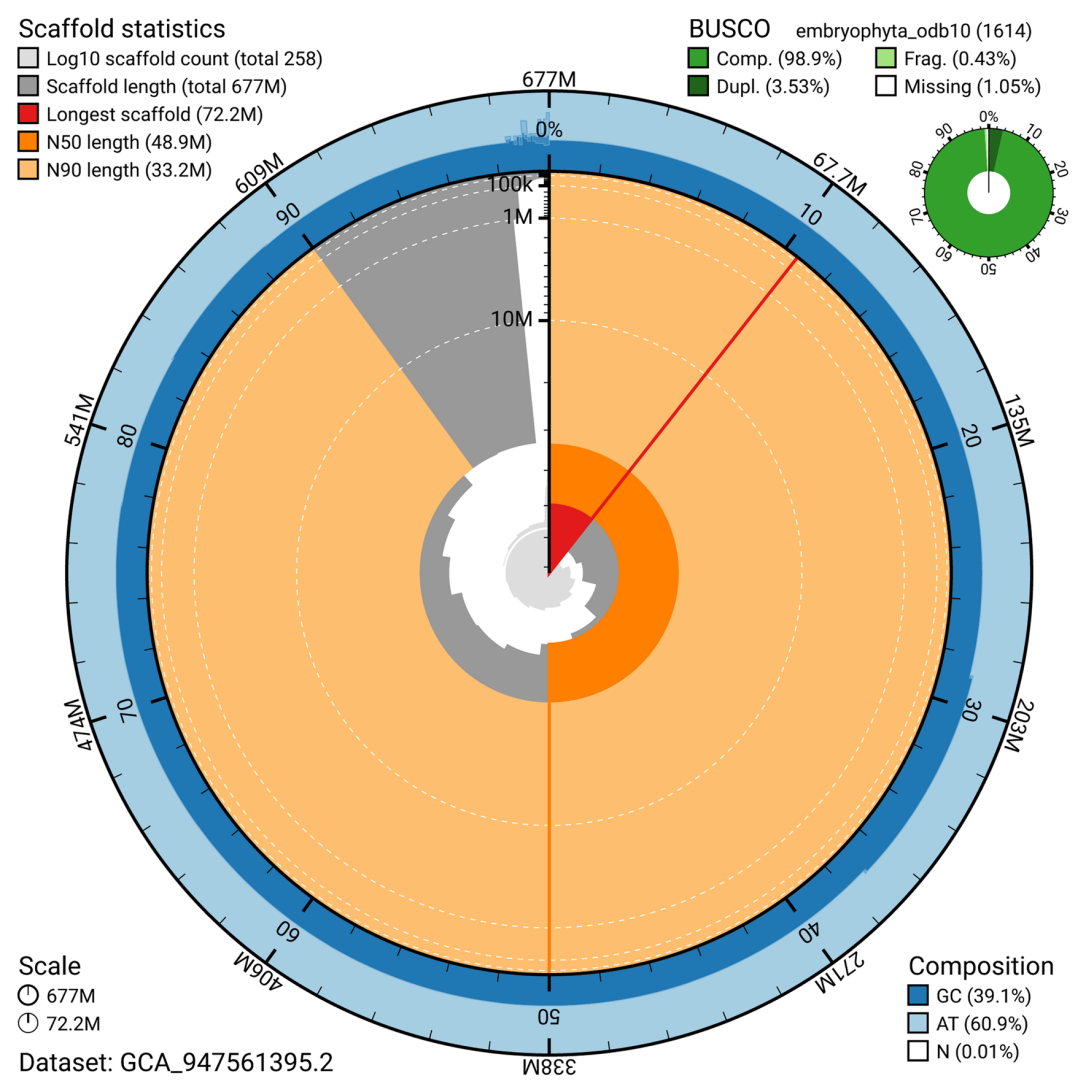
Genome assembly of *Buxus sempervirens*, drBuxSemp1.2: metrics. The BlobToolKit snail plot shows N50 metrics and BUSCO gene completeness. $BTK_SNAIL_LEG An interactive version of this figure is available at https://blobtoolkit.genomehubs.org/view/GCA_947561395.2/dataset/GCA_947561395.2/snail.

**Figure 3.**
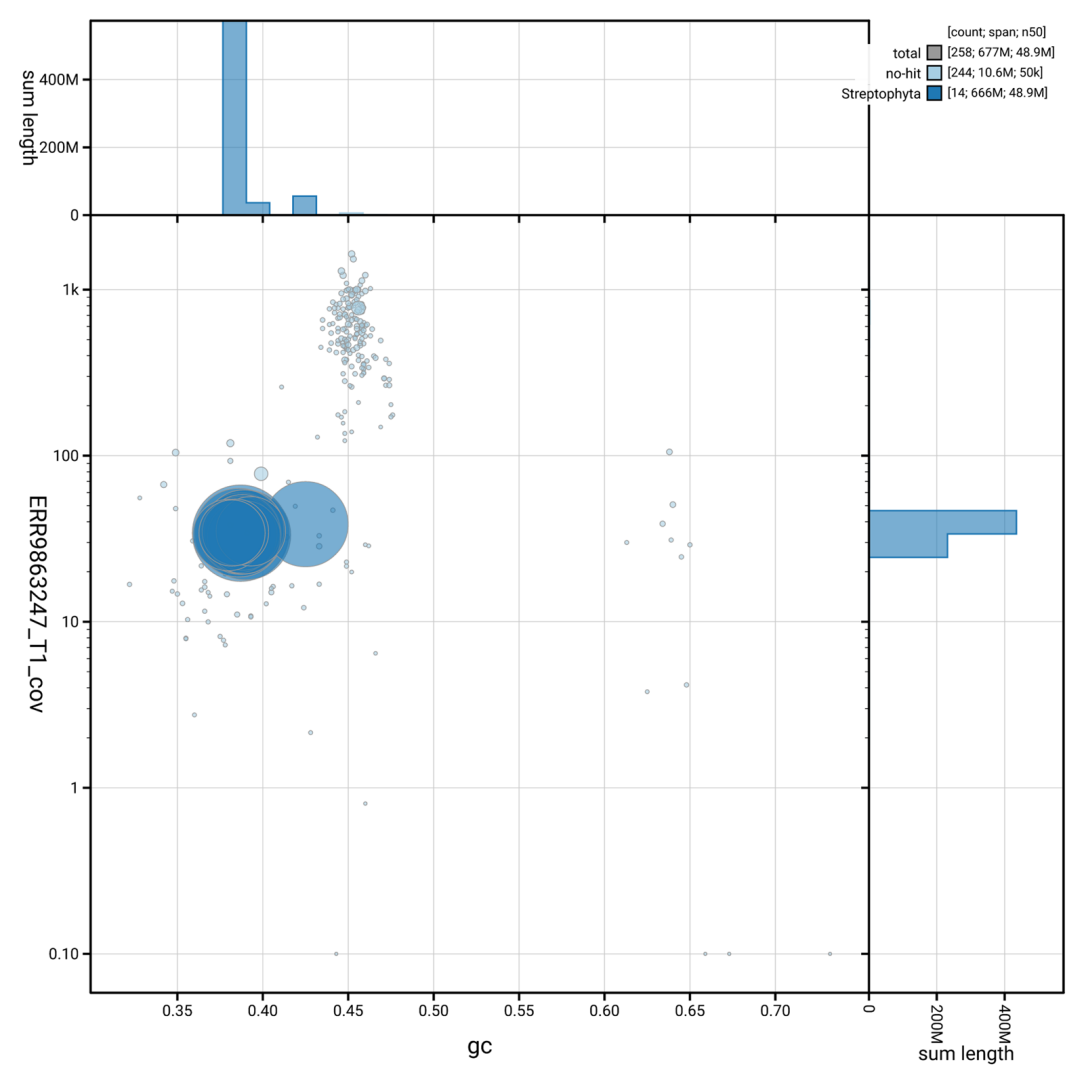
Genome assembly of *Buxus sempervirens*, drBuxSemp1.2: BlobToolKit GC-coverage plot. BlobToolKit GC-coverage plot showing sequence coverage (vertical axis) and GC content (horizontal axis). The circles represent scaffolds, with the size proportional to scaffold length and the colour representing phylum membership. The histograms along the axes display the total length of sequences distributed across different levels of coverage and GC content. An interactive version of this figure is available at https://blobtoolkit.genomehubs.org/view/GCA_947561395.2/dataset/GCA_947561395.2/blob.

**Figure 4.**
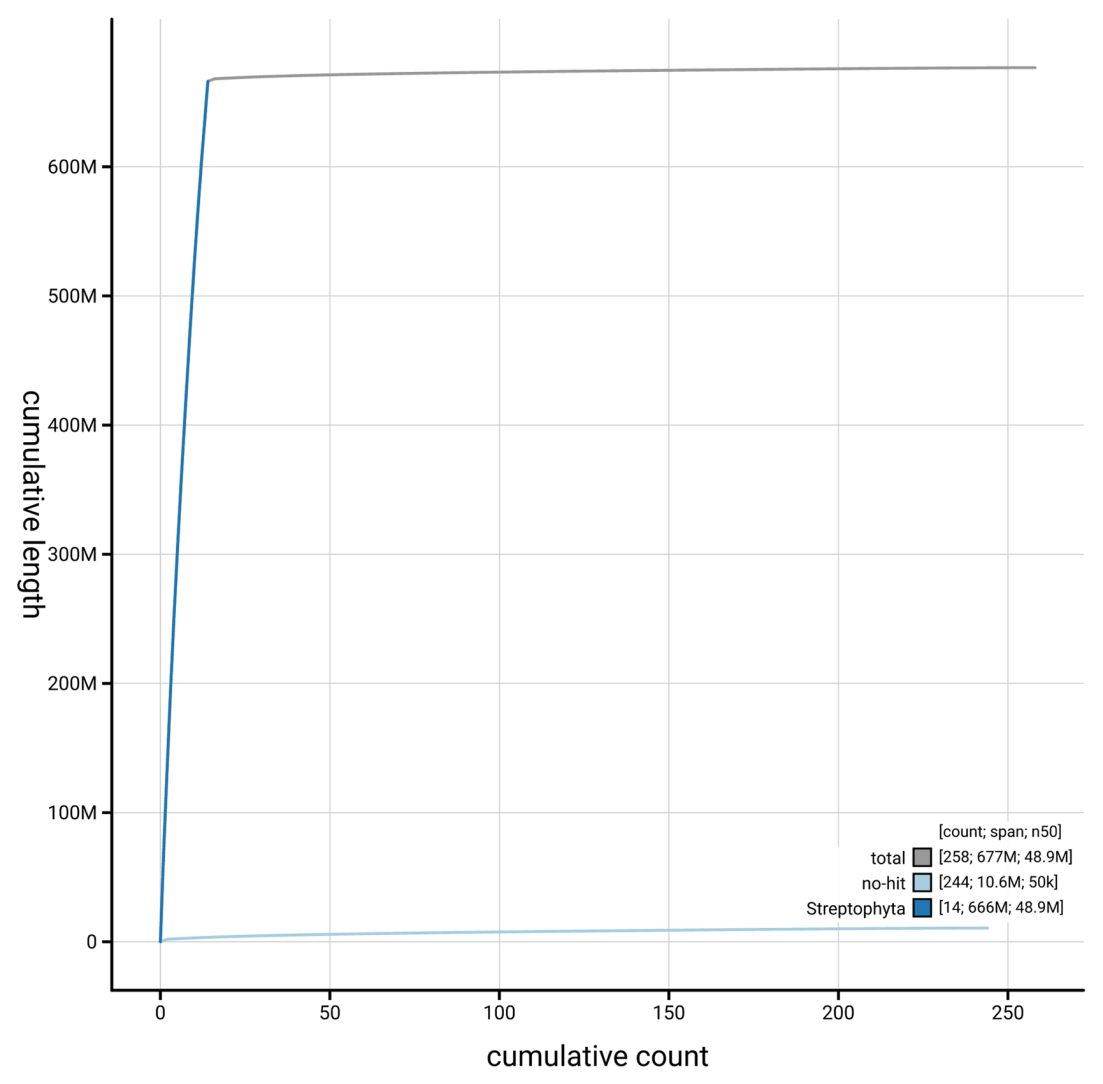
Genome assembly of *Buxus sempervirens*, drBuxSemp1.2: BlobToolKit cumulative sequence plot. The grey line shows cumulative length for all scaffolds. Coloured lines show cumulative lengths of scaffolds assigned to each phylum using the buscogenes taxrule. An interactive version of this figure is available at https://blobtoolkit.genomehubs.org/view/GCA_947561395.2/dataset/GCA_947561395.2/cumulative.

The snail plot in Figure 2 provides a summary of the assembly statistics, indicating the distribution of scaffold lengths and other assembly metrics. Figure 3 shows the distribution of scaffolds by GC proportion and coverage. Figure 4 presents a cumulative assembly plot, with separate curves representing different scaffold subsets assigned to various phyla, illustrating the completeness of the assembly.

**Figure 5.**
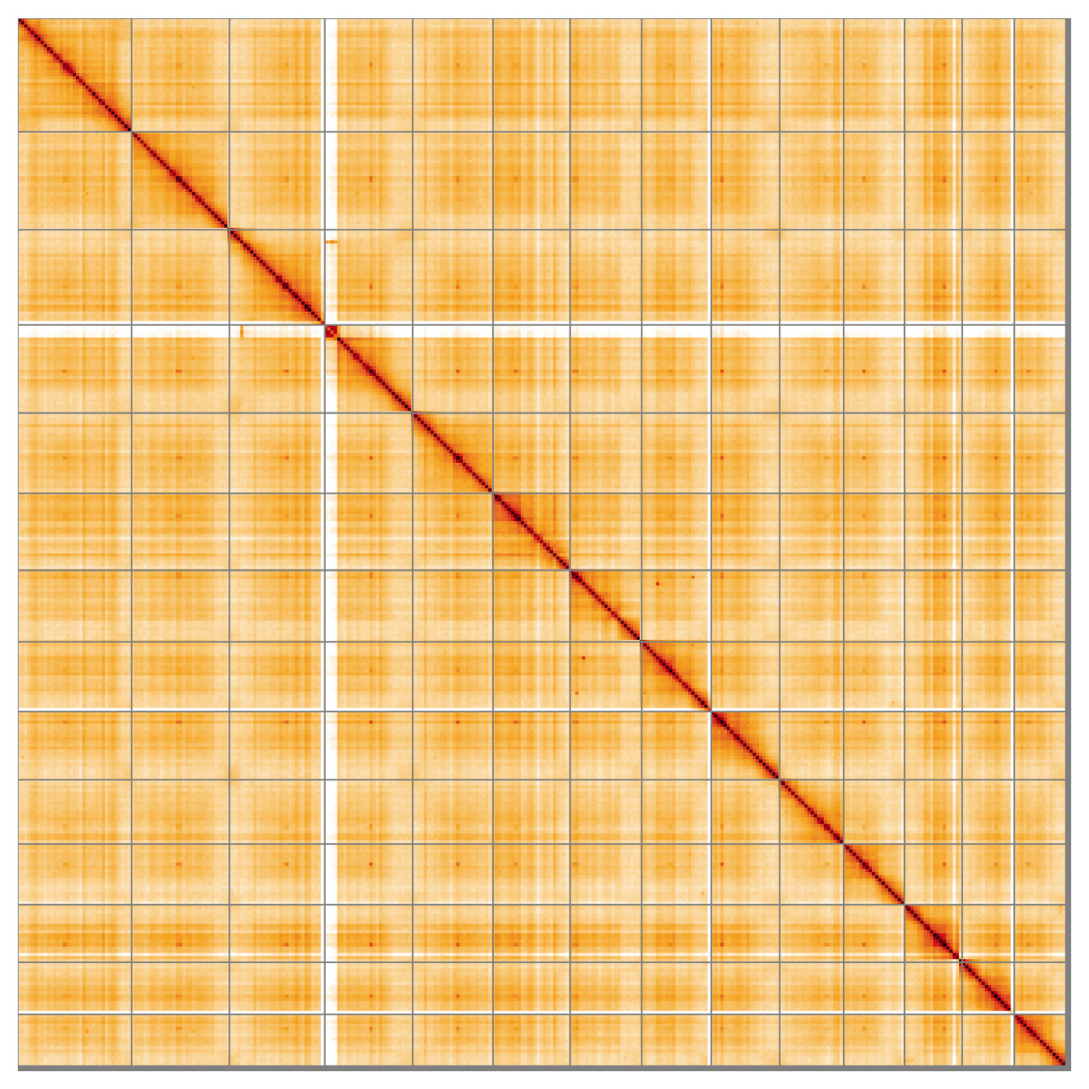
Genome assembly of *Buxus sempervirens*, drBuxSemp1.2: Hi-C contact map of the drBuxSemp1.2 assembly, visualised using HiGlass. Chromosomes are shown in order of size from left to right and top to bottom. An interactive version of this figure may be viewed at https://genome-note-higlass.tol.sanger.ac.uk/l/?d=YyD8vzoNQWW1Abn51N4vKw.

Most (98.66%) of the assembly sequence was assigned to 14 chromosomal-level scaffolds. Chromosome-scale scaffolds confirmed by the Hi-C data are named in order of size ( Figure 5; Table 3). During manual curation it was noted that the order and orientation of scaffolds are uncertain on chromosome 6 in the region 3.26–7.58 Mb. While not fully phased, the assembly deposited is of one haplotype. Contigs corresponding to the second haplotype have also been deposited. The mitochondrial and plastid genomes were also assembled and can be found as contigs within the multifasta file of the genome submission.

**Table 3.** Chromosomal pseudomolecules in the genome assembly of *Buxus sempervirens*, drBuxSemp1.

INSDC accession	Name	Length (Mb)	GC%
OX387182.1	1	72.17	38.5
OX387183.1	2	62.34	39.0
OX387184.1	3	60.58	38.5
OX387185.1	4	55.89	42.5
OX387186.1	5	50.97	38.5
OX387187.1	6	48.88	39.0
OX387188.1	7	45.64	39.0
OX387189.1	8	44.03	38.5
OX387190.1	9	43.63	39.0
OX387191.1	10	40.76	39.0
OX387192.1	11	38.62	38.0
OX387193.1	12	36.46	39.5
OX387194.1	13	33.19	38.5
OX387195.1	14	32.97	38.0
OZ124166.1	Pltd	0.15	38.0
OZ124158.1	MT1	0.71	45.5
OZ124159.1	MT2	0.02	46.0
OZ124160.1	MT3	0.09	45.5
OZ124161.1	MT4	0.03	45.5
OZ124162.1	MT5	0.03	45.0
OZ124163.1	MT6	0.05	45.5
OZ124164.1	MT7	0.02	44.0
OZ124165.1	MT8	0.03	46.0

The final assembly has a Quality Value (QV) of 63.5 and *k*-mer completeness of 100.0%. BUSCO (v5.4.3) analysis using the embryophyta_odb10 reference set ( *n* = 1,614) indicated a completeness score of 99.0% (single = 95.5%, duplicated = 3.5%).

Metadata for specimens, BOLD barcode results, spectra estimates, sequencing runs, contaminants and pre-curation assembly statistics are given at https://links.tol.sanger.ac.uk/species/4002.

## Methods

### Sample acquisition, DNA barcoding and genome size estimation

A specimen of *Buxus sempervirens* (specimen ID KDTOL10106, ToLID drBuxSemp1) was cultivated in the Royal Botanic Gardens Kew Conservation Area (310). The plant was originally picked by hand from Box Hill (latitude 51.47, longitude –0.31) on 2020-09-08. The specimen was collected and identified by Maarten J. M. Christenhusz (MC9100) and preserved by freezing at –80 °C. The herbarium voucher associated with the sequenced plant is K001400701 and is deposited in the herbarium of RBG Kew.

The initial species identification was verified by an additional DNA barcoding process according to the framework developed by Twyford *et al.* (2024). Part of the plant specimen was preserved in silica gel desiccant ( Chase & Hills, 1991). A DNA extraction from the dried plant was amplified by PCR for standard barcode markers, with the amplicons sequenced and compared to public sequence databases including GenBank and the Barcode of Life Database (BOLD). The barcode sequences for this specimen are openly available on BOLD ( Ratnasingham & Hebert, 2007). Following whole genome sequence generation, DNA barcodes were also used alongside the initial barcoding data for sample tracking through the genome production pipeline at the Wellcome Sanger Institute ( Twyford *et al.*, 2024). The standard operating procedures for the Darwin Tree of Life barcoding are available on protocols.io ( Beasley *et al.*, 2023).

The genome size was estimated by flow cytometry using the fluorochrome propidium iodide and following the ‘one-step’ method as outlined in Pellicer *et al.* (2021). For this species, the General Purpose Buffer (GPB) supplemented with 3% PVP and 0.08% (v/v) beta-mercaptoethanol was used for isolation of nuclei ( Loureiro *et al.*, 2007), and the internal calibration standard was *Petroselinum crispum* ‘Champion Moss Curled’ with an assumed 1C-value of 2,200 Mb ( Obermayer *et al.*, 2002).

### Nucleic acid extraction

The workflow for high molecular weight (HMW) DNA extraction at the Wellcome Sanger Institute (WSI) Tree of Life Core Laboratory includes a sequence of core procedures: sample preparation and homogenisation, DNA extraction, fragmentation and purification. Detailed protocols are available on protocols.io ( Denton *et al.*, 2023). The drBuxSemp1 sample was prepared for DNA extraction by weighing and dissecting it on dry ice ( Jay *et al.*, 2023). Leaf tissue was homogenised by cryogenic bead beating ( Jackson & Howard, 2023b). HMW DNA was extracted using the Plant Organic HMW gDNA Extraction method ( Jackson & Howard, 2023a). HMW DNA was sheared into an average fragment size of 12–20 kb in a Megaruptor 3 system ( Bates *et al.*, 2023). Sheared DNA was purified by solid-phase reversible immobilisation, using AMPure PB beads to eliminate shorter fragments and concentrate the DNA ( Oatley *et al.*, 2023). The concentration of the sheared and purified DNA was assessed using a Nanodrop spectrophotometer, Qubit Fluorometer and Qubit dsDNA High Sensitivity Assay kit. Fragment size distribution was evaluated by running the sample on the FemtoPulse system.

RNA was extracted from leaf tissue of drBuxSemp1 in the Tree of Life Laboratory at the WSI using the RNA Extraction: Automated MagMax™ *mir*Vana protocol ( do Amaral *et al.*, 2023). The RNA concentration was assessed using a Nanodrop spectrophotometer and a Qubit Fluorometer using the Qubit RNA Broad-Range Assay kit. Analysis of the integrity of the RNA was done using the Agilent RNA 6000 Pico Kit and Eukaryotic Total RNA assay.

### Hi-C preparation

Leaf tissue of the drBuxSemp1 sample was processed at the WSI Scientific Operations core, using the Arima-HiC v2 kit. Tissue (stored at –80 °C) was fixed, and the DNA crosslinked using a TC buffer with 22% formaldehyde. After crosslinking, the tissue was homogenised using the Diagnocine Power Masher-II and BioMasher-II tubes and pestles. Following the kit manufacturer's instructions, crosslinked DNA was digested using a restriction enzyme master mix. The 5’-overhangs were then filled in and labelled with biotinylated nucleotides and proximally ligated. An overnight incubation was carried out for enzymes to digest remaining proteins and for crosslinks to reverse. A clean up was performed with SPRIselect beads prior to library preparation.

### Library preparation and sequencing

Library preparation and sequencing were performed at the WSI Scientific Operations core. Pacific Biosciences HiFi circular consensus DNA sequencing libraries were prepared using the PacBio Express Template Preparation Kit v2.0 (Pacific Biosciences, California, USA) as per the manufacturer's instructions. The kit includes the reagents required for removal of single-strand overhangs, DNA damage repair, end repair/A-tailing, adapter ligation, and nuclease treatment. Library preparation also included a library purification step using AMPure PB beads (Pacific Biosciences, California, USA) and size selection step to remove templates shorter than 3 kb using AMPure PB modified SPRI. DNA concentration was quantified using the Qubit Fluorometer v2.0 and Qubit HS Assay Kit and the final library fragment size analysis was carried out using the Agilent Femto Pulse Automated Pulsed Field CE Instrument and gDNA 165kb gDNA and 55kb BAC analysis kit. Samples were sequenced using the Sequel IIe system (Pacific Biosciences, California, USA). The concentration of the library loaded onto the Sequel IIe was between 40–135 pM. The SMRT link software, a PacBio web-based end-to-end workflow manager, was used to set-up and monitor the run, as well as perform primary and secondary analysis of the data upon completion.

For Hi-C library preparation, DNA was fragmented to a size of 400 to 600 bp using a Covaris E220 sonicator. The DNA was then enriched, barcoded, and amplified using the NEBNext Ultra II DNA Library Prep Kit following manufacturers’ instructions. The Hi-C sequencing was performed using paired-end sequencing with a read length of 150 bp on an Illumina NovaSeq 6000 instrument.

Poly(A) RNA-Seq libraries were constructed using the NEB Ultra II RNA Library Prep kit, following the manufacturer’s instructions. RNA sequencing was performed on the Illumina NovaSeq 6000 instrument.

### Genome assembly, curation and evaluation

***Assembly***

The HiFi reads were first assembled using Hifiasm ( Cheng *et al.*, 2021) with the --primary option. Haplotypic duplications were identified and removed using purge_dups ( Guan *et al.*, 2020). The Hi-C reads were mapped to the primary contigs using bwa-mem2 ( Vasimuddin *et al.*, 2019). The contigs were further scaffolded using the provided Hi-C data ( Rao *et al.*, 2014) in YaHS ( Zhou *et al.*, 2023) using the --break option for handling potential misassemblies. The scaffolded assemblies were evaluated using Gfastats ( Formenti *et al.*, 2022), BUSCO ( Manni *et al.*, 2021) and MERQURY.FK ( Rhie *et al.*, 2020). The organellar genomes were assembled using OATK ( Zhou, 2023).

***Curation***

The assembly was checked for contamination and corrected using the gEVAL system ( Chow *et al.*, 2016) as described previously ( Howe *et al.*, 2021). Manual curation was primarily conducted using PretextView ( Harry, 2022), with additional insights provided by JBrowse2 ( Diesh *et al.*, 2023) and HiGlass ( Kerpedjiev *et al.*, 2018). Scaffolds were visually inspected and corrected as described by Howe *et al.* (2021). Any identified contamination, missed joins, and mis-joins were corrected, and duplicate sequences were tagged and removed. The process is documented at https://gitlab.com/wtsi-grit/rapid-curation (article in preparation).

***Evaluation of final assembly***

The final assembly was post-processed and evaluated with the three Nextflow ( Di Tommaso *et al.*, 2017) DSL2 pipelines “sanger-tol/readmapping” ( Surana *et al.*, 2023a), “sanger-tol/genomenote” ( Surana *et al.*, 2023b), and “sanger-tol/blobtoolkit” ( Muffato *et al.*, 2024). The pipeline sanger-tol/readmapping aligns the Hi-C reads with bwa-mem2 ( Vasimuddin *et al.*, 2019) and combines the alignment files with SAMtools ( Danecek *et al.*, 2021). The sanger-tol/genomenote pipeline transforms the Hi-C alignments into a contact map with BEDTools ( Quinlan & Hall, 2010) and the Cooler tool suite ( Abdennur & Mirny, 2020), which is then visualised with HiGlass ( Kerpedjiev *et al.*, 2018). It also provides statistics about the assembly with the NCBI datasets ( Sayers *et al.*, 2024) report, computes *k*-mer completeness and QV consensus quality values with FastK and MERQURY.FK, and a completeness assessment with BUSCO ( Manni *et al.*, 2021).

The sanger-tol/blobtoolkit pipeline is a Nextflow port of the previous Snakemake Blobtoolkit pipeline ( Challis *et al.*, 2020). It aligns the PacBio reads with SAMtools and minimap2 ( Li, 2018) and generates coverage tracks for regions of fixed size. In parallel, it queries the GoaT database ( Challis *et al.*, 2023) to identify all matching BUSCO lineages to run BUSCO ( Manni *et al.*, 2021). For the three domain-level BUSCO lineage, the pipeline aligns the BUSCO genes to the Uniprot Reference Proteomes database ( Bateman *et al.*, 2023) with DIAMOND ( Buchfink *et al.*, 2021) blastp. The genome is also split into chunks according to the density of the BUSCO genes from the closest taxonomically lineage, and each chunk is aligned to the Uniprot Reference Proteomes database with DIAMOND blastx. Genome sequences that have no hit are then chunked with seqtk and aligned to the NT database with blastn ( Altschul *et al.*, 1990). All those outputs are combined with the blobtools suite into a blobdir for visualisation.

The genome assembly and evaluation pipelines were developed using the nf-core tooling ( Ewels *et al.*, 2020), use MultiQC ( Ewels *et al.*, 2016), and make extensive use of the Conda package manager, the Bioconda initiative ( Grüning *et al.*, 2018), the Biocontainers infrastructure ( da Veiga Leprevost *et al.*, 2017), and the Docker ( Merkel, 2014) and Singularity ( Kurtzer *et al.*, 2017) containerisation solutions. Table 4 contains a list of relevant software tool versions and sources.

**Table 4.** Software tools: versions and sources.

Software tool	Version	Source
BEDTools	2.30.0	https://github.com/arq5x/bedtools2
BLAST	2.14.0	ftp://ftp.ncbi.nlm.nih.gov/blast/executables/blast+/
BlobToolKit	4.3.7	https://github.com/blobtoolkit/blobtoolkit
BUSCO	5.4.3 and 5.5.0	https://gitlab.com/ezlab/busco
bwa-mem2	2.2.1	https://github.com/bwa-mem2/bwa-mem2
Cooler	0.8.11	https://github.com/open2c/cooler
DIAMOND	2.1.8	https://github.com/bbuchfink/diamond
fasta_windows	0.2.4	https://github.com/tolkit/fasta_windows
FastK	427104ea91c78c3b8b8b49f1a7d6bbeaa869ba1c	https://github.com/thegenemyers/FASTK
gEVAL	N/A	https://geval.org.uk/
Gfastats	1.3.6	https://github.com/vgl-hub/gfastats
GoaT CLI	0.2.5	https://github.com/genomehubs/goat-cli
Hifiasm	0.16.1-r375	https://github.com/chhylp123/hifiasm
HiGlass	44086069ee7d4d3f6f3f0012569789ec138f42b84 aa44357826c0b6753eb28de	https://github.com/higlass/higlass
Merqury.FK	d00d98157618f4e8d1a9190026b19b471055b22e	https://github.com/thegenemyers/MERQURY.FK
MultiQC	1.14, 1.17, and 1.18	https://github.com/MultiQC/MultiQC
NCBI Datasets	15.12.0	https://github.com/ncbi/datasets
Nextflow	23.04.0-5857	https://github.com/nextflow-io/nextflow
OATK	1	https://github.com/c-zhou/oatk
PretextView	0.2	https://github.com/sanger-tol/PretextView
purge_dups	1.2.3	https://github.com/dfguan/purge_dups
samtools	1.16.1, 1.17, and 1.18	https://github.com/samtools/samtools
sanger-tol/genomenote	1.1.1	https://github.com/sanger-tol/genomenote
sanger-tol/readmapping	1.2.1	https://github.com/sanger-tol/readmapping
Seqtk	1.3	https://github.com/lh3/seqtk
Singularity	3.9.0	https://github.com/sylabs/singularity
YaHS	yahs-1.1.91eebc2	https://github.com/c-zhou/yahs

### Wellcome Sanger Institute – Legal and Governance

The materials that have contributed to this genome note have been supplied by a Darwin Tree of Life Partner. The submission of materials by a Darwin Tree of Life Partner is subject to the **‘Darwin Tree of Life Project Sampling Code of Practice’**, which can be found in full on the Darwin Tree of Life website here. By agreeing with and signing up to the Sampling Code of Practice, the Darwin Tree of Life Partner agrees they will meet the legal and ethical requirements and standards set out within this document in respect of all samples acquired for, and supplied to, the Darwin Tree of Life Project.

Further, the Wellcome Sanger Institute employs a process whereby due diligence is carried out proportionate to the nature of the materials themselves, and the circumstances under which they have been/are to be collected and provided for use. The purpose of this is to address and mitigate any potential legal and/or ethical implications of receipt and use of the materials as part of the research project, and to ensure that in doing so we align with best practice wherever possible. The overarching areas of consideration are:

•   Ethical review of provenance and sourcing of the material

•   Legality of collection, transfer and use (national and international)

Each transfer of samples is further undertaken according to a Research Collaboration Agreement or Material Transfer Agreement entered into by the Darwin Tree of Life Partner, Genome Research Limited (operating as the Wellcome Sanger Institute), and in some circumstances other Darwin Tree of Life collaborators.

## Data Availability

European Nucleotide Archive: *Buxus sempervirens*. Accession number PRJEB53560; https://identifiers.org/ena.embl/PRJEB53560 ( Wellcome Sanger Institute, 2022). The genome sequence is released openly for reuse. The *Buxus sempervirens* genome sequencing initiative is part of the Darwin Tree of Life (DToL) project. All raw sequence data and the assembly have been deposited in INSDC databases. The genome will be annotated using available RNA-Seq data and presented through the Ensembl pipeline at the European Bioinformatics Institute. Raw data and assembly accession identifiers are reported in Table 1.

## Author information

Members of the Royal Botanic Gardens Kew Genome Acquisition Lab are listed here: https://doi.org/10.5281/zenodo.12625079.

Members of the Plant Genome Sizing collective are listed here: https://doi.org/10.5281/zenodo.7994306.

Members of the Darwin Tree of Life Barcoding collective are listed here: https://doi.org/10.5281/zenodo.12158331.

Members of the Wellcome Sanger Institute Tree of Life Management, Samples and Laboratory team are listed here: https://doi.org/10.5281/zenodo.12162482.

Members of Wellcome Sanger Institute Scientific Operations: Sequencing Operations are listed here: https://doi.org/10.5281/zenodo.12165051.

Members of the Wellcome Sanger Institute Tree of Life Core Informatics team are listed here: https://doi.org/10.5281/zenodo.12160324.

Members of the Tree of Life Core Informatics collective are listed here: https://doi.org/10.5281/zenodo.12205391.

Members of the Darwin Tree of Life Consortium are listed here: https://doi.org/10.5281/zenodo.4783558.
