# Supplementary material for: The genome sequence of common box, Buxus sempervirens L. (Buxaceae)
Source: Wellcome Open Res. 2026 Feb 18;9:683. Originally published 2024 Nov 20. [Version 2] doi: 10.12688/wellcomeopenres.23267.2 (PMC12976612; doi:10.12688/wellcomeopenres.23267.2)
Supplement: Peer review history

# Reviewer report: Reviewer response for version 2

**DOI:** 10.21956/wellcomeopenres.28653.r148710

**Published:** 2026-03-14

**Authors:** Roberto Biello

**Affiliations:** 1 University of Ferrara, Ferrara, Italy

The authors present a high-quality, chromosome-level genome assembly for common box, *Buxus sempervirens*, generated using PacBio HiFi and Hi-C sequencing. This assembly represents a valuable genomic resource for future studies on the evolution and biology of this important plant species. The assembly meets high-quality standards, supported by strong BUSCO completeness and k-mer-based metrics. The methods are clearly described, and all associated data are publicly available.

Are sufficient details of methods and materials provided to allow replication by others?

Yes

Is the rationale for creating the dataset(s) clearly described?

Yes

Are the datasets clearly presented in a useable and accessible format?

Yes

Are the protocols appropriate and is the work technically sound?

Yes

Reviewer Expertise:

Evolutionary biology, Genomics

I confirm that I have read this submission and believe that I have an appropriate level of expertise to confirm that it is of an acceptable scientific standard.

# Reviewer report: Reviewer response for version 2

**DOI:** 10.21956/wellcomeopenres.28653.r148183

**Published:** 2026-03-10

**Authors:** Harpreet Kaur

**Affiliations:** 1 University of Minnesota, St. Paul, Minnesota, USA

The authors have addressed all of my comments satisfactorily. I appreciate the authors for their careful revisions and responses.

Are sufficient details of methods and materials provided to allow replication by others?

Yes

Is the rationale for creating the dataset(s) clearly described?

Yes

Are the datasets clearly presented in a useable and accessible format?

Yes

Are the protocols appropriate and is the work technically sound?

Partly

Reviewer Expertise:

Genetics and Genomics

I confirm that I have read this submission and believe that I have an appropriate level of expertise to confirm that it is of an acceptable scientific standard.

# Reviewer report: Reviewer response for version 1

**DOI:** 10.21956/wellcomeopenres.25645.r117029

**Published:** 2025-01-23

**Authors:** Harpreet Kaur

**Affiliations:** 1 University of Minnesota, St. Paul, Minnesota, USA

The manuscript titled *“* *The Genome Sequence of Common Box, Buxus sempervirens L. (* *Buxaceae)”* presents the genome assembly of the common box plant. The study provides sufficient details for replication; however, I have the following queries and suggestions for improvement:

1. **Inclusion of RNA Extraction Data**:
  Why is RNA extraction data mentioned in the methodology and data availability when it is not utilized in the study? If RNA data is available, why not use it for annotating the genome assembly and include the results in the manuscript? Incorporating annotation results could enhance the overall assembly quality, as annotation often impacts BUSCO coverage (generally reducing it). This would further build confidence in the assembly.
2. **Coverage Calculation**:
  How was the genome assembly coverage calculated? The reported coverage does not seem to match the provided values of 31-fold and 165-fold for PacBio HiFi and Hi-C data, respectively, based on dividing the total read length (size of read x total number of reads) by the estimated genome size (890 MB). Could you provide more clarity on this calculation?
3. **Genome Size Estimation and Repetitive Sequences**:
  Including k-mer-based genome size estimation and repetitive sequence analysis would strengthen the manuscript. These analyses provide valuable insights into genome characteristics and further support the robustness of the assembly.

Are sufficient details of methods and materials provided to allow replication by others?

Yes

Is the rationale for creating the dataset(s) clearly described?

Yes

Are the datasets clearly presented in a useable and accessible format?

Yes

Are the protocols appropriate and is the work technically sound?

Partly

Reviewer Expertise:

Alfalfa Genetics and Genomics

I confirm that I have read this submission and believe that I have an appropriate level of expertise to confirm that it is of an acceptable scientific standard, however I have significant reservations, as outlined above.

# Response

**Published:** 2026-02-04

**Authors:** Tree of Life Team Sanger

**Affiliations:** Wellcome Trust Sanger Institute, UK

We thank the reviewer for these comments. **1. RNA sequencing data**

 RNA sequencing data are listed in the Methods and Data availability sections because they have been generated and are publicly available for reuse. As with other Darwin Tree of Life genome notes, we do not present genome annotation results in this manuscript. Annotation of DToL assemblies is carried out centrally by Ensembl at EMBL–EBI, using standardised pipelines applied consistently across taxa. **2. Genome coverage calculation**

 Genome size and coverage estimates were derived from *k*-mer analysis using GenomeScope2.0, rather than from the flow cytometric genome size estimate. We have clarified this in the Methods and Results sections and now explicitly describe the GenomeScope2.0 approach used. **3. Genome size estimation and repetitive sequence content**

 We agree that *k*-mer–based analyses provide useful context. Information on GenomeScope2.0 genome size estimation and repeat content has now been added to the manuscript to better characterise the genome and support the assembly metrics presented. Tree of Life team, Wellcome Sanger Institute

# Reviewer report: Reviewer response for version 1

**DOI:** 10.21956/wellcomeopenres.25645.r112789

**Published:** 2024-12-27

**Authors:** Parimalan Rangan

**Affiliations:** 1 ICAR-National Bureau of Plant Genetic Resources, New Delhi, India

Although the manuscript details out the genome assembly for *Buxus sempervirens*, few more details can be added.  For example, the authors may perform structural annotation to add value to the assembly.  Additionally, they can also include functional annotation for it will be of more utility for other fellow researchers to study on its biological information with special reference to the pathways involved in it.

Are sufficient details of methods and materials provided to allow replication by others?

Yes

Is the rationale for creating the dataset(s) clearly described?

Yes

Are the datasets clearly presented in a useable and accessible format?

Yes

Are the protocols appropriate and is the work technically sound?

Yes

Reviewer Expertise:

Transcriptomics, genomics, molecular biology

I confirm that I have read this submission and believe that I have an appropriate level of expertise to confirm that it is of an acceptable scientific standard.
